# Neurocognitive deficits in HIV-positive patients—two case reports: Revising current AANTF guidelines in view of recent revelation of new neurocognitive symptoms

**DOI:** 10.4103/0019-5545.31585

**Published:** 2006

**Authors:** V.N. Vahia, Tejas Bhojraj, Dean A. Creado

**Affiliations:** * Honorary Psychiatrist and Professor, Seth G.S. Medical College and and R.N. Cooper Hospital, Mumbai; ** Student (III MBBS Final Semester), Seth G.S. Medical College and and R.N. Cooper Hospital, Mumbai; *** Lecturer, Seth G.S. Medical College and and R.N. Cooper Hospital, Mumbai

**Keywords:** Neurocognitive deficit (NCD), HIV testing, AANTF (American Academy of Neurology Task Force) guidelines

## Abstract

Certain organic antecedents such as fever, weight loss, diarrhoea and systemic infections often present with neurocognitive deficits (NCDs). However, routine HIV screening is not done in such cases. HIV can present with psychiatric and neurocognitive symptoms as highlighted in the two cases given below.

Case 1, a housewife, had been exhibiting altered behaviour following a low-grade fever over the past 3 weeks, associated with muttering to self, talking irrelevantly, would wander away from home, had decreased sleep, loss of appetite, and neglected self-care. She had displayed impulsivity by jumping into a well. On admission, the patient was mute, lethargic and the cerebrospinal fluid (CSF) tested positive for cryptococcus. Her human immunodeficiency virus (HIV) status was positive.

Case 2, a housewife, presented with one-month history of muttering to self, increased irritability, aggressive on minimal provocation, decreased sleep, loss of appetite, and suspiciousness towards family members. On provisional diagnosis of schizophrenia, the patient was started on low-dose antipsychotic drugs, which showed minimal improvement. There was a distinct slowness in her movements and she progressively lost weight. Routine investigations were normal but her HIV status was positive.

It has recently come to light that HIV infection also presents with subtle manifestations of the central nervous system (CNS), which are distinct from NCD and, if harnessed, could enhance diagnostic sensitivity and reduce the ‘asymptomatic period’. Hence HIV testing is recommended in such cases.

## INTRODUCTION

Human immunodeficiency virus (HIV) infection is reported to manifest with neurocognitive deficits (NCDs) as symptoms. HIV may also present with other mild deficits such as apathy, sensorimotor and gait disturbances.^1^ Intriguingly, chemo-therapy for HIV induces parallel NCD-like symptoms^2^,^3^ in conjunction with certain strikingly unique ones^4^ in a degree sufficient to impair life.^5^ The correlation between NCDs and CD4 counts in HIV is affected by age, which exemplifies the multifactorial nature of symptoms in the central nervous system (CNS). An area deserving concern is the surfacing of new NCDs such as verbal fluency,^6^ verbal and spatial memory^7^ and apathy^8^ with the potential to enhance diagnosis. Some tests, such as neurometric and ERP records, predate the appearance of conventional symptoms, and hence have a potential to diagnose HIV earlier.^9^ Bornstein *et al.*^10^ showed the precedence, in time, of cognitive deficits over conventional AIDS symptoms. This may point to a more sensitive marker for AIDS *per se* rather than CD4 counts. These symptoms are not included in the current AANTF guidelines despite their superior sensitivity in detecting HIV, albeit there is lack of data regarding their specificity.

Memory deficit (yet another example) is a symptom often reported with HIV and is believed to be the cause of NCD.^7^ Auditory working memory dysfunction is just one more instance of the protean CNS effects of HIV. Hence, the following two case reports are used to illustrate the need to include such symptoms in the current guidelines (AANTF) for NCD.

1. HIV screening is warranted by organic antecedents such as fever, weight loss and diarrhoea occurring in not only patients with NCDs, but also in those with memory loss,^7^ apathy,^8^ ERP deficits and neurometric deficits.^9^ Additions must be made to the existent diagnostic criteria, of the newly revealed sensitive CNS markers of HIV (see also the list of suggested changes).
2. Criteria must be appropriately changed to prevent confounding factors such as
  —highly active antiretroviral therapy (HAART), non-nucleoside reverse transcriptase inhibitor (NNRTI), efavirenz-induced NCD.^2^–^5^
  —Hepatitis C and encephalitis-induced NCD.^11^

## CASE 1

A 30-year-old housewife was hospitalized for self-inflicted 70% burns. The patient had been exhibiting altered behaviour following a low-grade fever over the past 3 weeks. She was muttering to self, talking irrelevantly, would wander away from home, had decreased sleep, loss of appetite and neglected self-care. She had displayed impulsivity by jumping into a well.

On admission, the patient was conscious, did not cooperate for mental status examination, had a fixed gaze and did not respond to oral commands. She reacted to painful stimuli. There was no show of recognition towards family members. She had blunted affect. There was no posturing, tics, stereotypes or mannerisms. On investigation, routine blood count, EEG, CT scan were within normal limits. CSF tested positive for cryptococcus. HIV status was positive. In the ward, the patient was mute, lethargic and had to be persuaded to do daily chores. No altered behaviour similar to the presenting complaints was seen.

She was diagnosed with encephalopathy due to dual infection by HIV and cryptococcus. She was started on flucanozole and showed minimal improvement.

## CASE 2

A 32-year-old housewife presented with one-month history of muttering to self, increased irritability, aggressiveness on minimal provocation, decreased sleep, loss of appetite, and suspiciousness towards family members.

On provisional diagnosis of schizophrenia, the patient was started on low-dose antipsychotic drugs. Minimal improvement in behaviour was seen. Subsequent visits showed the patient to be unmotivated. There was a distinct slowness in her movements and she had progressive loss of weight. Routine investigations were normal but her HIV status was positive.

## DISCUSSION

The AANTF guidelines mentioned below have to be revised in view of the following data.

1. Neurocognitive deficits:
  - Antiretroviral therapy (ART), HAART, NNRTI, efavirenz cause significant NCDs.^2^–^5^
  - A recent study linked improvement of NCDs such as verbal fluency to HAART, raising the issue of its conflicting effects on NCDs.^5^
2. Depression caused by HIV, independently and with HIV, impairs neurocognitive functions. NCD findings have to be normalized accordingly.^12^
3. Revelation of markers such as impairment on spatial and verbal working memory, apathy, auditory defects, sensori-motor loss and odour disturbances.^7^,^8^,^13^–^15^
4. Dementia is an easily quantifiable and standardized marker; many studies have successfully proved its validity in providing formal diagnostic criteria.^16^
5. Tests such as the Hiscock test of memory can be used to detect feigning of NCD for disability benefits. The scoring system used in memory tests such as the WMS-R (Wechsler) have to be revised to correct for malingering exhibited by subjects seeking benefits.^17^,^18^
6. A study at Harvard discovered the usefulness of ERP recordings and standard neurometric testing in diagnosing HIV in ‘asymptomatic’ patients—one more example of acuity these new CNS markers can lend to the recent NCD guidelines.^9^
7. Bornstein *et al.*^10^ and Wilkie *et al.*^19^ have separately proved the early appearance of cognitive deficits in asymptomatic cases of HIV.
8. Different criteria for old and young age groups are mandated by studies showing different levels of (i) motor–cognitive impairment, and (ii) RTs (reaction times).^20^
9. CD4 criteria for patients with apathy have to be different from the criteria for those with other neurocognitive symptoms, as warranted by differing correlations in both symptomatic groups with CD4 counts. Thus, criteria for correlation between apathy and cognitive function, between CD4 count and apathy, and between CD4 count and cognitive function need to be revised.^8^
10. Similar NCD markers and symptoms also occur in encephalitis and hepatitis C, both of which are possible confounding factors. Viral loads in CSF can be useful in excluding encephalitis.^11^,^21^

To summarize, clinical sensitivity for identifying HIV-related syndrome is contingent upon awareness of the symptoms of NCD. Clinical sensitivity can—in view of the recently uncovered more sensitive symptoms (e.g. ERP)—be increased beyond merely detecting NCD. Bornstein *et al.* and Wilkie *et al.* have demonstrated early cognitive impairment in asymptomatic cases, thus increasing diagnostic sensitivity of HIV directly in proportion to the earliness of appearance of these symptoms.

In view of the possibility of a major outbreak or cataclysm that epidemiologists have been cautioning us against, measures have to be taken to develop strategies for early identification (by early markers such as ERP records, neurometry, etc.). Infrastructure for effective management of NCD by drugs such as methylphenidate^22^ has to be mobilized. Further support for these recommendations are based on the following:

- A study by Bryan *et al.* in Indian truck drivers used NCD for diagnosing HIV cases, thus substantiating the claim of a purported correlation between HIV and NCD.^23^
- Inter-rater reliability tests^24^ for the criteria of NCD lent significance to the diagnostic value of the criteria by demonstrating its consistent reproducibility in varied clinical settings.

Hence NCD can be relied upon in the new strategy for awareness and containment of HIV. Drugs such as methyl-phenidate cure NCD symptoms in HIV,^22^ which are hypothesized to arise from working memory deficits. An efficient way to treat NCD would be to use HAART in appropriate doses to ameliorate HIV and NCD, given its recently proven effectiveness in the latter case. Such drugs if made available on a large scale can help contain these symptoms. But HAART and drugs such as NNRTI, protease inhibitors, efavirenz also cause NCD.^2^–^5^ Hence HIV testing and history of ART or drugs is essential in NCD. ART and efavirenz must be used cautiously as a cured post-traumatic stress disorder (PTSD) can be reactivated.

With these caveats, a full-fledged screening programme for HIV in patients with NCD should be instituted. The basic guidelines should be used as these have been vindicated in diverse clinical settings, but should be made more specific and adept at earlier detection by including symptoms mentioned above.
